# Contribution of coronal vertebral and IVD wedging to Cobb angle changes in adolescent idiopathic scoliosis during growth

**DOI:** 10.1186/s12891-022-05863-z

**Published:** 2022-10-10

**Authors:** Wing Ki Cheung, Jason Pui Yin Cheung

**Affiliations:** Department of Orthopaedics and Traumatology, The University of Hong Kong, Professorial Block, 5th Floor, 102 Pokfulam Road, Pokfulam, Hong Kong SAR, China

**Keywords:** Adolescent idiopathic scoliosis, Disc, Wedging, Deformity

## Abstract

**Study design:**

Prospective study

**Background:**

Vertebral and intervertebral disc (IVD) wedging are often seen in patients with adolescent idiopathic scoliosis (AIS). However, the relationship between wedging and curve progression, and the change of wedging before bracing to final weaning is unknown. The aim of this study was to investigate the pattern and sequence of vertebral and IVD wedging development, and to determine the relationship between the change of wedging and curve progression in AIS during growth.

**Methods:**

This was a prospective study of 32 AIS females with right-sided thoracic curves and/or left-sided lumbar curves who completed brace treatment. They were classified into progression and non-progression groups. Vertebral and IVD wedging were calculated for each spinal segment. The wedging pattern was first identified and then used to determine the sequence of wedging development. Percentage change in the sum of wedging during growth was calculated and compared.

**Results:**

The sum of vertebral wedging for both groups was 2.4° to 8.7° more than that of IVD wedging in the thoracic spine but 8.7° to 17.7° less in the lumbar spine. Out of the 20 curves assessed, 5 thoracic curves and 1 lumbar curve developed vertebral wedging before IVD wedging, and 3 thoracic curves and 4 lumbar curves had the opposite pattern. The progression group had larger increases in sum of vertebral (40%) and IVD (28.6%) wedging as compared to the non-progression group (both 16.7%). A significant difference in wedging between the first and the latest visits was found in the progression group only (*p* < 0.05).

**Conclusion:**

Pattern and sequence of vertebral and IVD wedging were related to the location of the curve rather than the presence of curve progression. Progressed curves were associated with increased wedging during growth.

**Level of evidence:**

II

## Introduction

Scoliosis is a spinal deformity with vertebral rotation in the transverse plane, thoracic hypokyphosis in the sagittal plane, and lateral deviation in the frontal plane [1, 2]. Patients who are identified during or after puberty are known as adolescent idiopathic scoliosis (AIS), which is the most common type of scoliosis with a prevalence rate of 1–3% in the US [3] and 3–4% in Hong Kong [4].

Since spinal deformities are associated with vertebral and intervertebral disc (IVD) wedging, the role of wedging in AIS is being investigated. A direct correlation between wedging and Cobb angle was found [5, 6]. However, this has been contested with inconsistent presence of wedging in early scoliosis [7, 8]. Vertebral wedging was also found in subjects without scoliosis [9]. Most studies calculated wedging at one time point during scoliosis. Without knowing how wedging changes with spinal curvature development, it is difficult to determine whether the wedging forms as part of the natural growth or with scoliosis. Longitudinal studies found wedging to be associated with the curve deformity[10, 11] but no changes in vertebral or IVD wedging occurred during curve progression [12, 13]. However, these studies did not investigate the effects of curve type, maturity level, and brace treatment, all of which may influence curve morphology and thus create difficulties for comparison. Hence, the relationship between wedging and curve progression is unclear.

No previous studies have investigated the change of wedging from before bracing to weaning brace treatment. The aim of this study was to investigate the pattern and sequence of wedging development in patients with AIS through growth, and to determine the relationship between the change of wedging and curve progression.

## Methods

### Patient population

Patients diagnosed with AIS were randomly recruited in our tertiary referral clinic during the period of May to December 2021. Ethics was approved by the local institutional review board (UW 21–501). Subjects who were females with a right-sided thoracic curve and/or left-sided lumbar curve, which was the most common curve type of AIS [14], and completed brace treatment were included. Clinical and radiological records were assessed from pre-brace till brace weaning. Patients were excluded if they exceeded Risser stage 2 at the first clinical visit as this violated the brace referral criteria set by the Scoliosis Research Society. Patients were classified into the progression group if their major curve (thoracic or thoracolumbar/lumbar) had more than 10 degrees Cobb angle progression [15], and the non-progression group for the rest. This larger cut-off was used to better study wedging patterns in patients with significant curve progression.

### Data collection and measurement

**Fig. 1:**
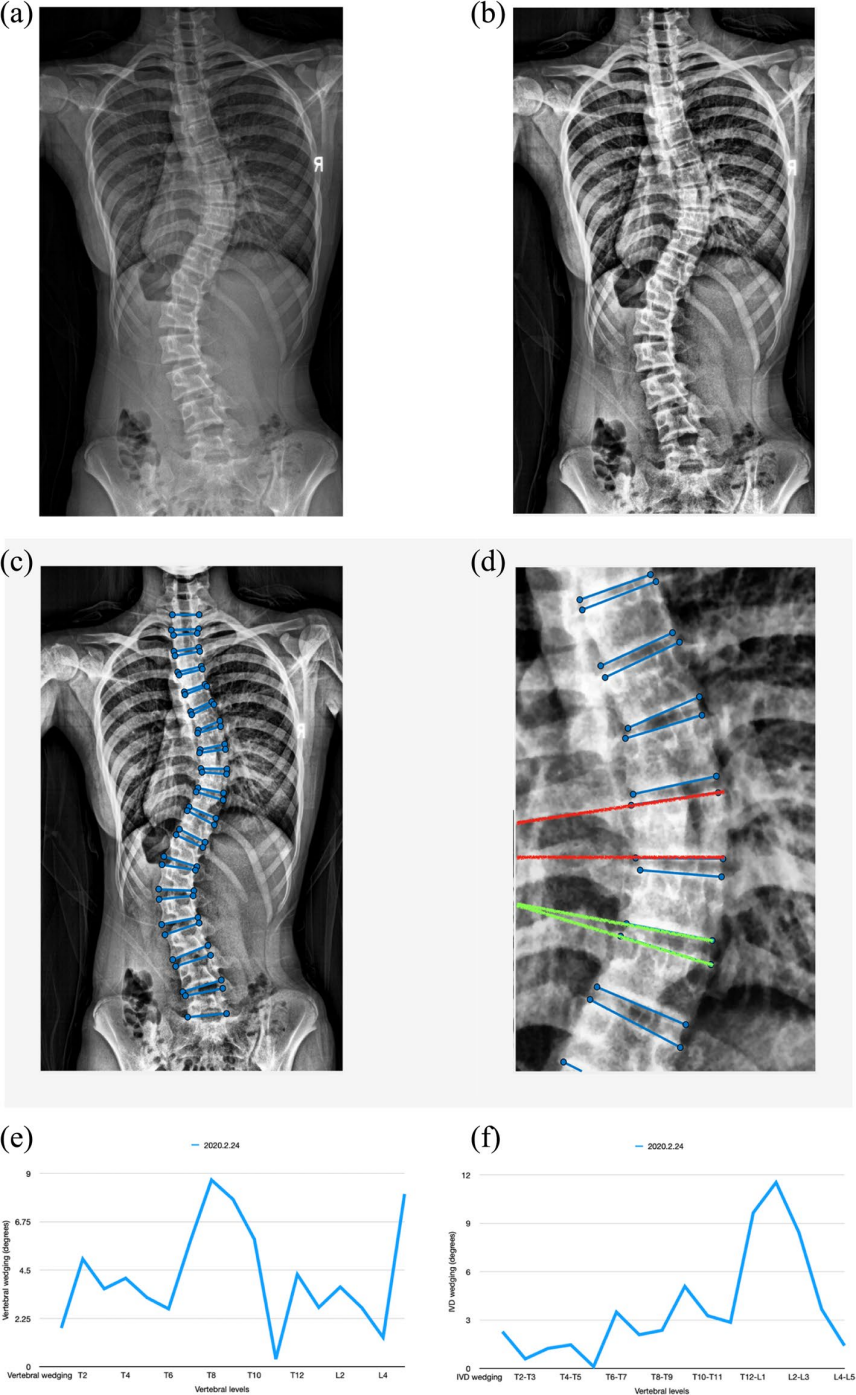
Steps in the measurement process. **a** Original X-ray. **b** After the X-ray was imported into MATLAB, a filter was used to enhance image contrast. **c** Lines across the superior and inferior endplates of each vertebra were drawn. **d** The lines passing through the superior and inferior endplate of the vertebra (red line) were used to calculate vertebral wedging while lines passing through the inferior endplate of the cranial vertebra and superior endplate of the caudal vertebra (green line) were used to calculate IVD wedging. **e** Vertebral and **f** IVD wedging were presented in a line graph to observe the change of wedging along the spine

Standing posterior-anterior biplanar radiographs of each recruited subject were collected from the first clinical visit to the latest (EOS® imaging, Paris, France). In-brace x-rays were excluded to avoid misinterpretation by the in-brace curve correction. Cobb angles and the Risser sign were measured. The interval between each scan was approximately six months. A MATLAB code was written to calculate the vertebral and IVD wedging by drawing a line across the superior and inferior endplates of each vertebra as shown in Fig. 1 (MATLAB, The MathWorks, Inc., US). The angle between the superior and inferior endplate of the particular vertebra was calculated to determine the degree of vertebral wedging. The angle between the inferior endplate of the cranial vertebra and superior endplate of the caudal vertebra calculated the intervening IVD wedging. Each follow-up was approximately 6 months apart.

### Pattern and sequence of wedging

**Fig. 2:**
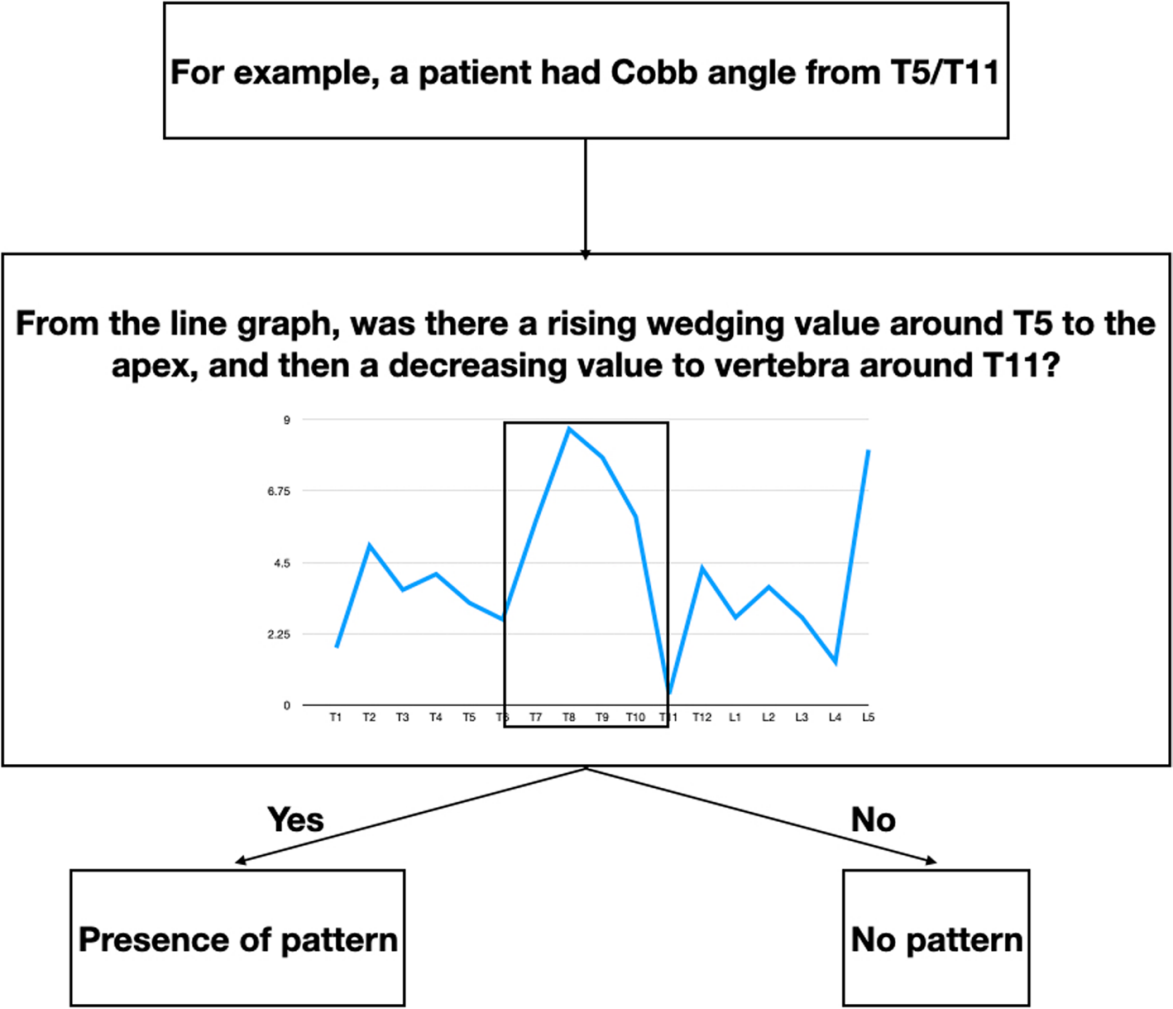
Pattern detection. If the wedging was maximum at the apex and decreased to the upper and lower vertebrae, this indicated the presence of a wedging pattern

Wedging was maximum at the apex and decreased to the upper and lower vertebrae as shown in Fig. 2 [6, 13, 16]. Line graphs of vertebral and IVD wedging at the first and latest clinical visit were assessed. The percentage of subjects having this pattern was calculated to determine whether the pattern presented at the early and later stages of scoliosis and to determine the relationship between this pattern and the location of the curve. To observe the change of pattern with time, differences in the percentage between two visits were calculated. Summation of all wedging for the entire thoracic spine, lumbar curve, and major curve was performed and compared to observe the contribution of wedging between two visits.

If a curve presented with both vertebral and IVD wedging patterns at the first clinical appointment, this curve was excluded from the assessment of the sequence of wedging development. For the remaining curves, line graphs of wedging from the first clinical visit to the latest were plotted in one graph which was used to observe whether the mentioned pattern developed in IVD first and followed by vertebrae or vice versa.

### Change of wedging during curve progression

**Fig. 3:**
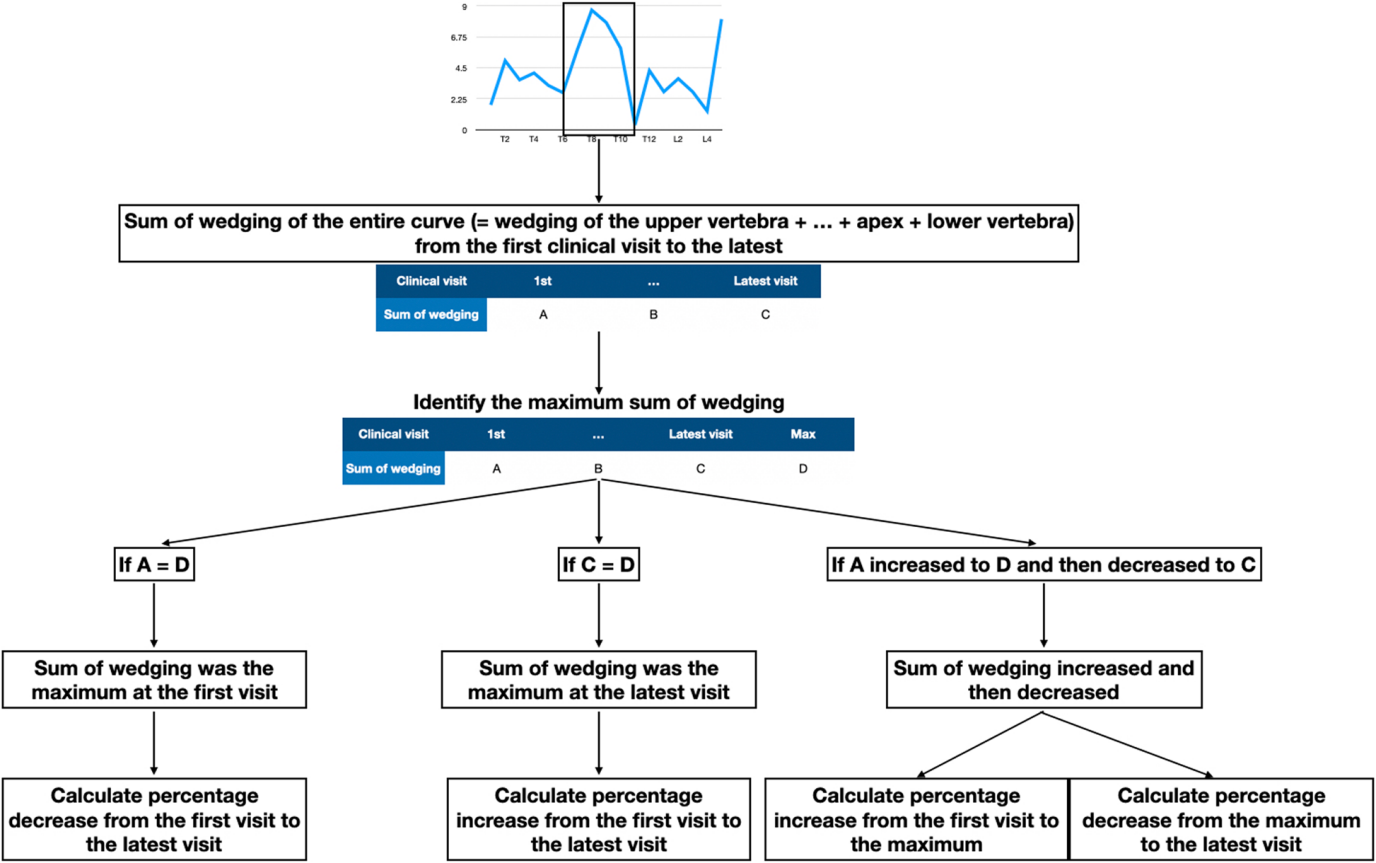
Curve classification. If the maximum sum of wedging was found at the first clinical visit, the percentage decrease from the first visit to the latest visit was calculated. If the maximum sum of wedging was found at the latest visit, the percentage increase from the first visit to the latest visit was calculated. If the maximum sum of wedging was found after the first visit and before the latest visit, the percentage increase from the first visit to the maximum sum and the percentage decrease from the maximum sum to the latest were calculated

In the progression group, a minor curve with less than 10 degrees increment in Cobb angle was excluded to ensure that all curves in the progression group had curve progression. In each subject, all wedging in the entire curve was summed and used to represent the change of wedging over time. The maximum sum of wedging which represented the maximum change during growth was first identified. The trend of the summation from the first consultation to the maximum and from the maximum to the latest visit was assessed. Each curve was identified into three conditions as shown in Fig. 3: the sum of wedging was maximum at the first visit; the sum of wedging increased during growth and was the maximum at the latest visit; and the trend of sum of wedging was increasing first till maximum summation and then decreased. After classification, percentage change was calculated. If the curve had the maximum sum of wedging at the first visit, the percentage decrease from the initial value to the latest value was calculated. If the curve had the maximum sum of wedging at the latest visit, the percentage increase from the initial sum to the latest sum was calculated. Lastly, if the sum of wedging increased and then decreased, the percentage change between the initial value and the maximum value, and between the maximum value and the latest value were calculated. The average percentage change in each group was calculated and compared. Line graphs of wedging from all visits were plotted on one graph to observe the change in wedging pattern between progressed and non-progressed curves.

### Statistical analysis

Statistical analysis was performed by Microsoft Excel (Microsoft, Redmond, WA, USA). F-test and two-tailed, paired t-test were used to compare the sum of wedging of the entire major curve between the first and the latest clinical visit with a significance level of *P* < 0.05. Mann–Kendall test was used to test whether the trend of wedging during growth was significant or not.

15 sets of radiographs were randomly selected to calculate the intra- and inter-rater reliability using Two-way random effects, absolute agreement, and single rater [17] analysis (Intraclass correlation, ICC[2,1]). ICC value less than 0.5 indicates poor reliability, between 0.5 to 0.75 is moderate reliability, between 0.75 to 0.9 suggests good reliability, and greater than 0.9 is excellent reliability [17, 18].

## Results

**Fig. 4:**
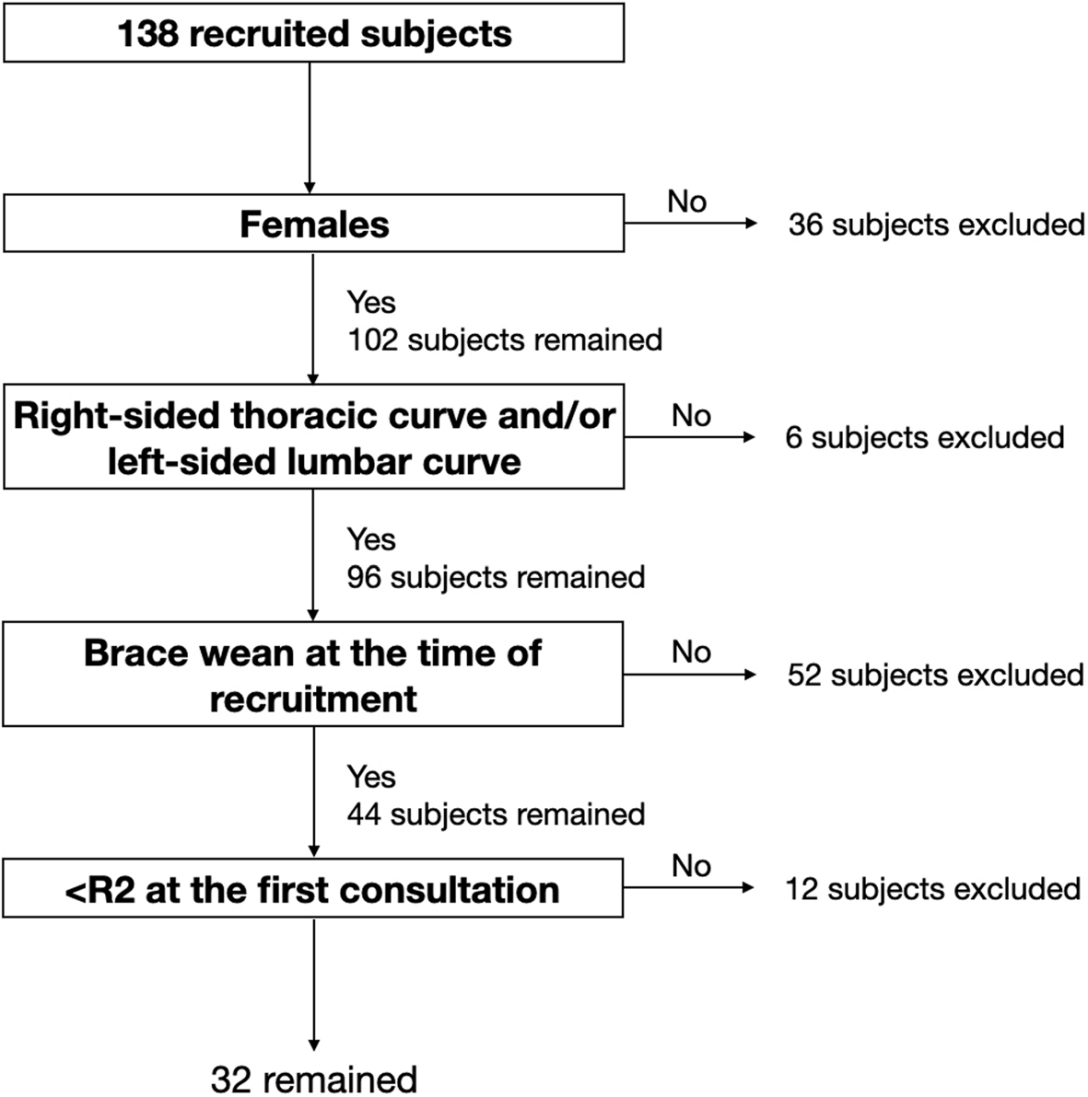
Recruitment flowchart

A total of 138 subjects were recruited and only 32 were eligible after exclusion as shown in Fig. 4. There were 12 subjects in the non-progression group and 20 subjects in the progression group. In the non-progression group, patients were at 12.8 ± 1.5 years old with major curve Cobb angle of 31.7 ± 8.1 degrees at the first consultation and aged 16.0 ± 2.0 years old with major curve Cobb angle of 34.5 ± 7.3 degrees at the latest visit. In the progression group, patients were aged 11.9 ± 1.5 years old with major curve Cobb angle of 23.8 ± 6.0 degrees at the first visit and were 17.2 ± 3.0 years old with major curve Cobb angle of 48.2 ± 11.2 degrees at the latest visit. The average brace wearing duration was 27.8 ± 9.7 months.

### Pattern of wedging

Maximum wedging at the apex which decreased to the upper and lower vertebrae in both vertebrae and IVD was observed for 80%, 75%, and 61.1% of the progression group and 92%, 66.7%, and 58.3% of the non-progression group for the major curve, thoracolumbar and lumbar curves, respectively at the first visit (Table 1). The percentage increased to 100%, 100% and 83.3% for the progression group and 100%, 83.3% and 66.7% for the non-progression group at the final visit. For the upper thoracic curve, 46.7% of the progression group and 25% of the non-progression group had this pattern in both vertebral and IVD wedging at the first visit and the percentage increased to 60% for the progression group and 62.5% for the non-progression group at the final visit.

**Table 1:** Pattern of wedging at the first and latest clinical visit

Percentage of subjects		Presence of the pattern in the major curve		Presence of pattern in upper thoracic curve		Presence of pattern in thoracolumbar curve		Presence of pattern in lumbar curve	
		First visit	Latest visit (change)	First visit	Latest visit (change)	First visit	Latest visit (change)	First visit	Latest visit (change)
Progression (*n* = 20)	Both	80%	100% ( +)	46.7%	60% ( +)	75%	100% ( +)	61.1%	83.3% ( +)
	VW	10%	0% (-)	53.3%	40% (-)	10%	0% (-)	5.6%	0% (-)
	IVDW	10%	0%(-)	/	/	15%	0% (-)	33.3%	16.7% (-)
Non-progression (*n* = 12)	Both	92%	100% ( +)	25%	62.5% ( +)	66.7%	83.3% ( +)	58.3%	66.7% ( +)
	VW	7.8%	0% (-)	75%	37.5% (-)	33.3%	16.7% (-)	/	/
	IVDW	/	/	/	/	/	/	41.7%	33.3% (-)

Note: *VW* in Vertebral wedging only, *IVDW* in IVD wedging onl, Both in both vertebral and IVD wedging

**Fig. 5:**
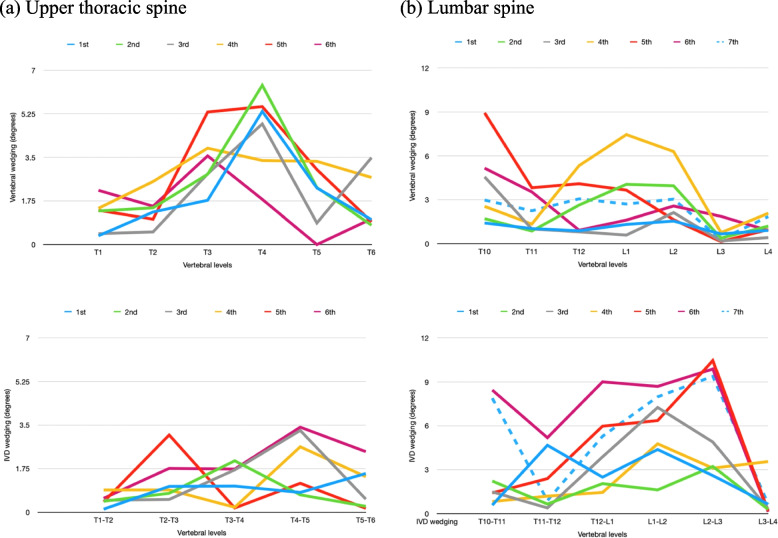
Relatively more vertebral wedging in the thoracic curve and more IVD wedging in the lumbar curve. **a** In the thoracic curve (T1-T6), a pattern of wedging was observed in the vertebral wedging for all visits but not in IVD wedging. The average sum of vertebral wedging was 14.1° while that of IVD wedging was 6.0°. **b** In the lumbar curve (T12-L4), a pattern of wedging was observed in IVD wedging for all visits but not in vertebral wedging. The average sum of vertebral wedging was 9.2° while that of IVD wedging was 16.4°

The average sum of vertebral wedging was 2.4° and 6.3° more than that of IVD wedging for progression and non-progression groups respectively in the thoracic spine at the first visit, and the difference between wedging increased to 8.7° and 7.1° at the latest visit (Table 2). The sum of IVD wedging in the lumbar curve was 8.7° and 10.9° more than that of vertebral wedging for progression and non-progression groups respectively and the differences also increased to 17.7° and 13.4° at the latest visit. For the major curve, the sum of IVD wedging (4.7° to 4.1°) was more than that of vertebral wedging (1.7° to 2.3°) for progression and non-progression groups from the first consultation to the latest. Figure 5 shows the line graphs of the wedging patterns and illustrates that more vertebral wedging occurs in the thoracic curve (a) and more IVD wedging occurs in the lumbar curve (b).

**Table 2:** Sum of wedging in the first and latest clinical visit

	Sum of vertebral wedging in						Sum of IVD wedging in (compared to the sum of vertebral wedging)					
	Thoracic curve		Lumbar curve		Major curve		Thoracic curve		Lumbar curve		Major curve	
	First visit (°)	Latest visit (°)	First visit (°)	Latest visit (°)	First visit (°)	Latest visit (°)	First visit (°)	Latest visit (°)	First visit (°)	Latest visit (°)	First visit (°)	Latest visit (°)
Progression (*n* = 20)	30.9 ± 10.8	45.7 ± 15.9	13.1 ± 5.3	14.5 ± 5.3	16.3 ± 6.2	25.9 ± 10.2	28.5 ± 8.5 (-2.4)	37.0 ± 10.6 (-8.7)	21.8 ± 7.3 (8.7)	32.2 ± 10.1 (17.7)	21.0 ± 6.0 (4.7)	30.0 ± 8.1 (4.1)
Non-progressio*n* (*n* = 12)	29.7 ± 7.2	32.5 ± 8.7	10.7 ± 4.3	11.6 ± 5.1	20.1 ± 8.2	20.9 ± 8.1	23.4 ± 9.2 (-6.3)	25.4 ± 9.0 (-7.1)	21.6 ± 8.1 (10.9)	25 ± 5.2 (13.4)	21.8 ± 8.3 (1.7)	23.2 ± 7.4 (2.3)

### Sequence of wedging

**Fig. 6:**
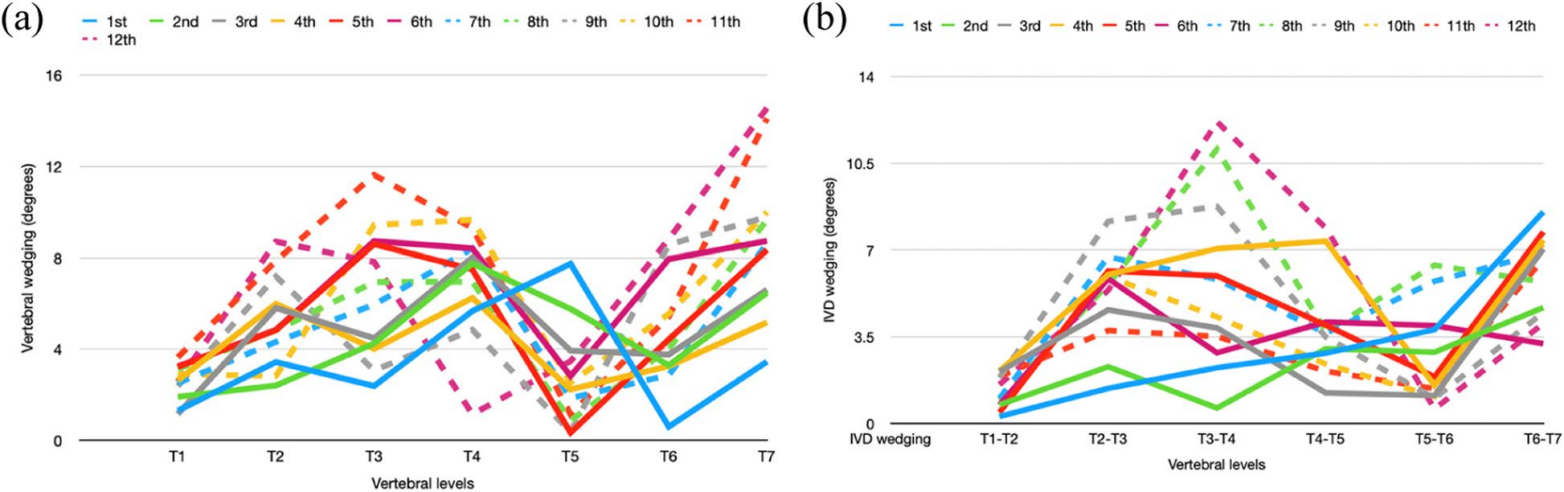
When vertebral wedging developed before IVD wedging. **a** The wedging pattern was presented at the first clinical visit. **b** At the first clinical visit, IVD wedging at the apex did not differ from others. It increased with time and was maximum at the fourth visit. Thus, vertebral wedging developed before IVD wedging

There were 65 curves and 45 of them were excluded because they had wedging in both vertebrae and IVD at the first clinical visit leaving 20 curves for analysis. 8 out of 20 curves had vertebral wedging only at the first visit and 6 out of 8 curves had both vertebral and IVD wedging at the latest visit. 2 out of 8 curves had vertebral wedging only from the first to the latest visit without IVD wedging developed and were found in the thoracic spine. Figure 6 illustrates when vertebral wedging occurs before IVD wedging and that it already occurs at the first clinical visit. At the fourth visit, IVD wedging was maximum at the apex and then decreased at the two ends. Thus, vertebral wedging developed first followed by IVD wedging.

**Fig. 7:**
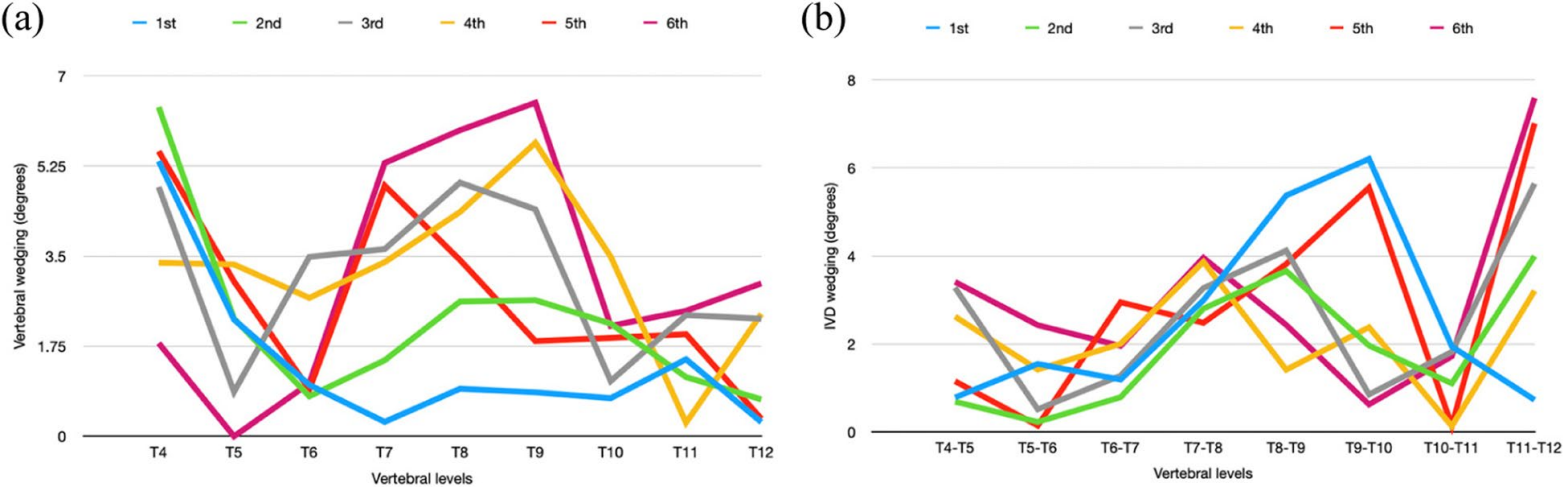
When IVD wedging developed before vertebral wedging. **a** Vertebral wedging at the apex did not differ from other vertebrae at the first clinical visit. It increased with time and was maximum at the third visit. **b** The wedging pattern already developed at the first clinical visit. Thus, IVD wedging developed before vertebral wedging

12 out of 20 curves had IVD wedging only at the first consultation and 7 out of 12 had both vertebral and IVD wedging at the latest visit. Among the 12 curves, 3 curves were found in the thoracic spine with vertebral wedging developed after IVD wedging. Other curves were found in the lumbar spine and 5 of them had IVD wedging only from the first consultation to the latest. Figure 7 shows that the wedging pattern was found in the IVD only at the first consultation and wedging was found in both vertebral and IVD at the third visit. Thus, in these cases, IVD wedging developed first and followed by vertebral wedging.

### Differences in wedging between the first and the latest visit

A significant difference was found between the sum of vertebral and IVD wedging of the major curve in the progression group between the first and latest visit (*p* < 0.05) but not in the non-progression group (*p* > 0.05) (Table 3).

**Table 3:** Significant difference in sum of wedging of the major curve between the first and latest visit

Significant difference in sum of wedging of the major curve between the first and latest visit		F-test	T-test
Progression (*n* = 20)	Vertebral Wedging	0.04*	0.001*
	IVD wedging	0.21	0.0003*
Non-progression (*n* = 12)	Vertebral Wedging	0.96	0.8
	IVD wedging	0.71	0.65

^*^denotes significance level *p* < 0.05

### Relationship between change in wedging and curve progression

**Fig. 8:**
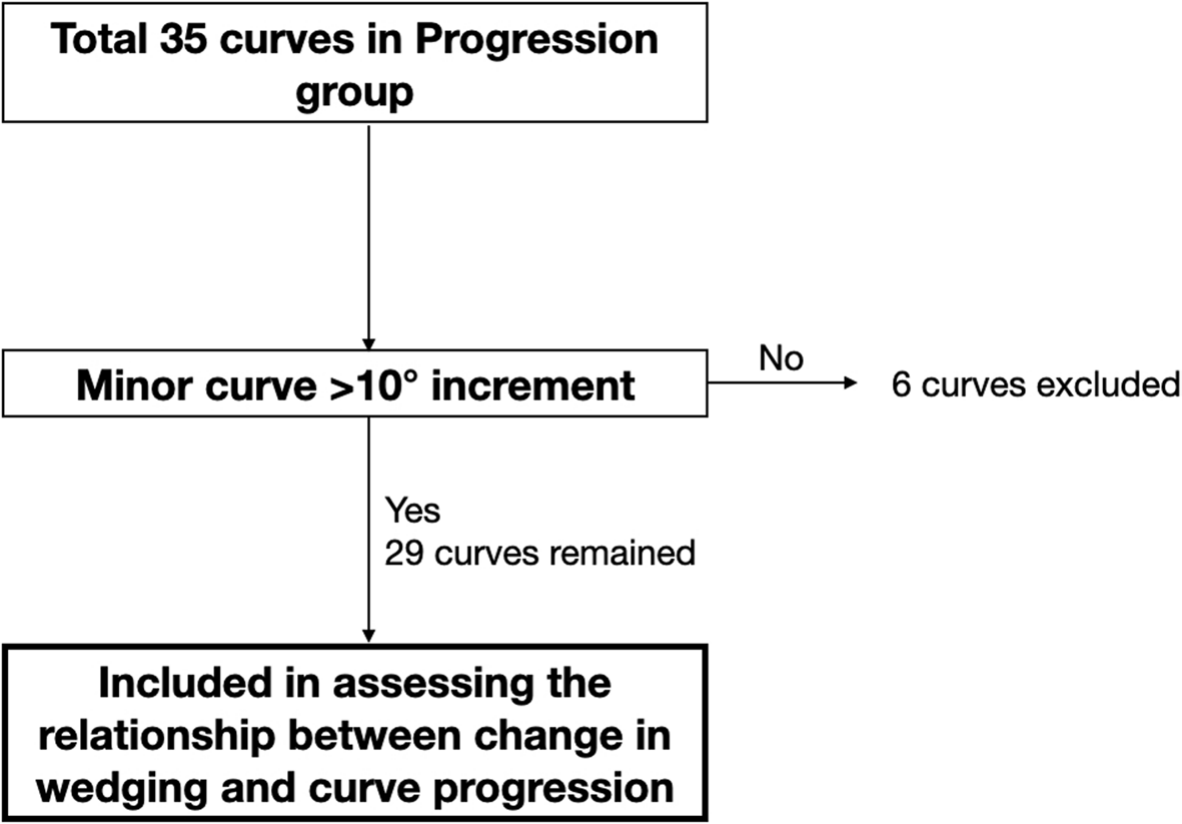
Curves were excluded from the Progression group when assessing the relationship between change in wedging and curve progression. 6 minor curves were excluded as the Cobb angle was increased less than 10°

A total of 35 curves were in the progression group and 6 of them were excluded as shown in Fig. 8. The non-progression group consisted of 24 curves. 48.6% and 62.9% of progressed curves and 62.5% and 66.7% of non-progressed curves had increasing sum of vertebral and IVD wedging respectively followed by a decrease (Table 4). However, the percentage increase from the initial wedging value to the maximum and the percentage decrease from the maximum wedging value to the latest in the progression group were 116% and 67.9% for vertebral wedging and 107% and 67.6% for IVD wedging respectively. These values were larger than that of the non-progression group (50.6% and 17.5% for vertebral wedging and 65.4% and 28.8% for IVD wedging respectively).

**Table 4:** Trend of wedging classification and the corresponding percentage change

Group	Wedging	Maximum sum of wedging at the first visit		Maximum sum of wedging at the latest visit		Sum of wedging increased and then decreased		
		Percentage of curve	Average percentage decrease from the first visit to the latest	Percentage of curve	Average percentage increase from the first visit to the latest	Percentage of curve	Average percentage increase from the initial value to the maximum	Average percentage decrease from the maximum to the latest value
Progression (*n* = 20)	Vertebral wedging	11.4%	32.9%	40%	72.7%	48.6%	116%	67.9%
	IVD wedging	8.6%	33.9%	28.6%	96.0%	62.9%	107%	67.6%
Non-progression (*n* = 12)	Vertebral wedging	20.8%	18.6%	16.7%	69.4%	62.5%	50.6%	17.5%
	IVD wedging	16.7%	27.0%	16.7%	45.8%	66.7%	65.4%	28.8%

**Fig. 9:**
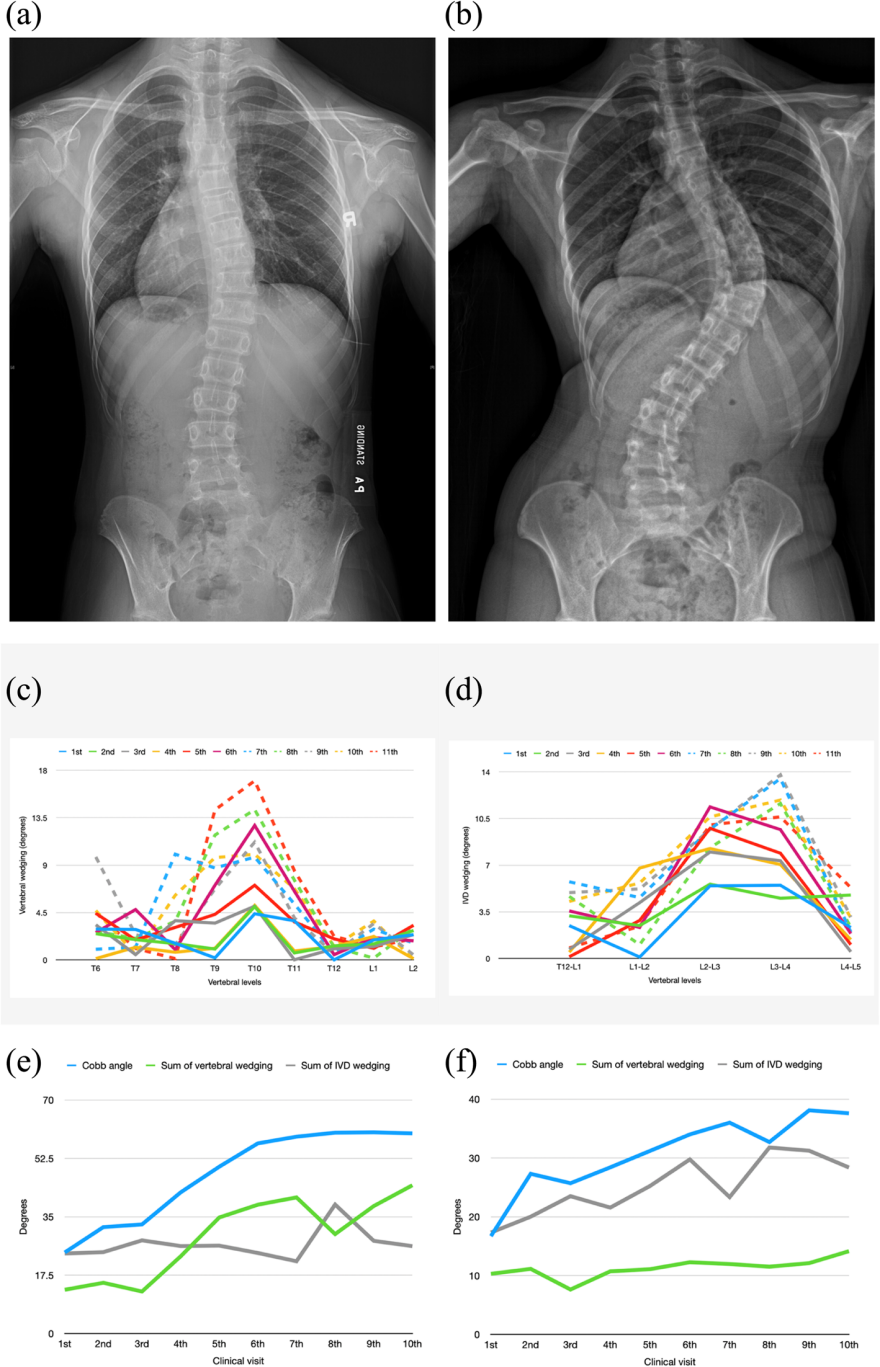
Progressed curves. **a** X-ray at the first clinical visit. T7/L1 = 24.3°. L1/L4 = 16.7°. **b** X-ray of the latest clinical visit. T7/L1 = 60°. L1/L5 = 37.6°. **c** Vertebral wedging of the thoracic curve. At the early stage of scoliosis, the wedging increased from T9 to T10 and then decreased to T11. The wedging increased from T8 to T10 and then decreased to T12. The wedging at the apex (T10) increased from 4.4° at the first clinical visit to 17.0° at the latest visit. **d** IVD wedging of the lumbar curve. The wedging at the apex (L2/L3) increased gradually from 5.4° at the first clinical visit to 10.0° at the latest visit. **e** Summation of the wedging value of the thoracic curve. Given that a gradual increase in wedging was found in vertebral wedging, the sum of the vertebral wedging was from 13.1° to 44.4°. **f** Summation of the wedging value of the lumbar curve. Given that a gradual increase in wedging was found in IVD wedging, the sum of the IVD wedging was from 17.3° to 28.4°

The progression group had 40% and 28.6% of curves which had the maximum sum of vertebral and IVD wedging respectively at the latest visit with 72.7% and 96.0% increase in the vertebral and IVD wedging respectively between the first and latest visit. However, the non-progression group only had 16.7% of curves having an increasing sum of wedging from the first to the latest visit with 69.4% and 45.8% increase in vertebral and IVD wedging respectively between the two visits. The percentage increase was larger in IVD wedging than vertebral wedging in the progressed group but the opposite was observed in the non-progressed group. From the line graph of wedging of the progressed curve, the wedging of the whole curve increased gradually and the increase in wedging was not only found at the apex but also in the adjacent vertebrae (Fig. 9). These changes in wedging were found in both vertebrae and IVD for a progressed curve.

**Fig. 10:**
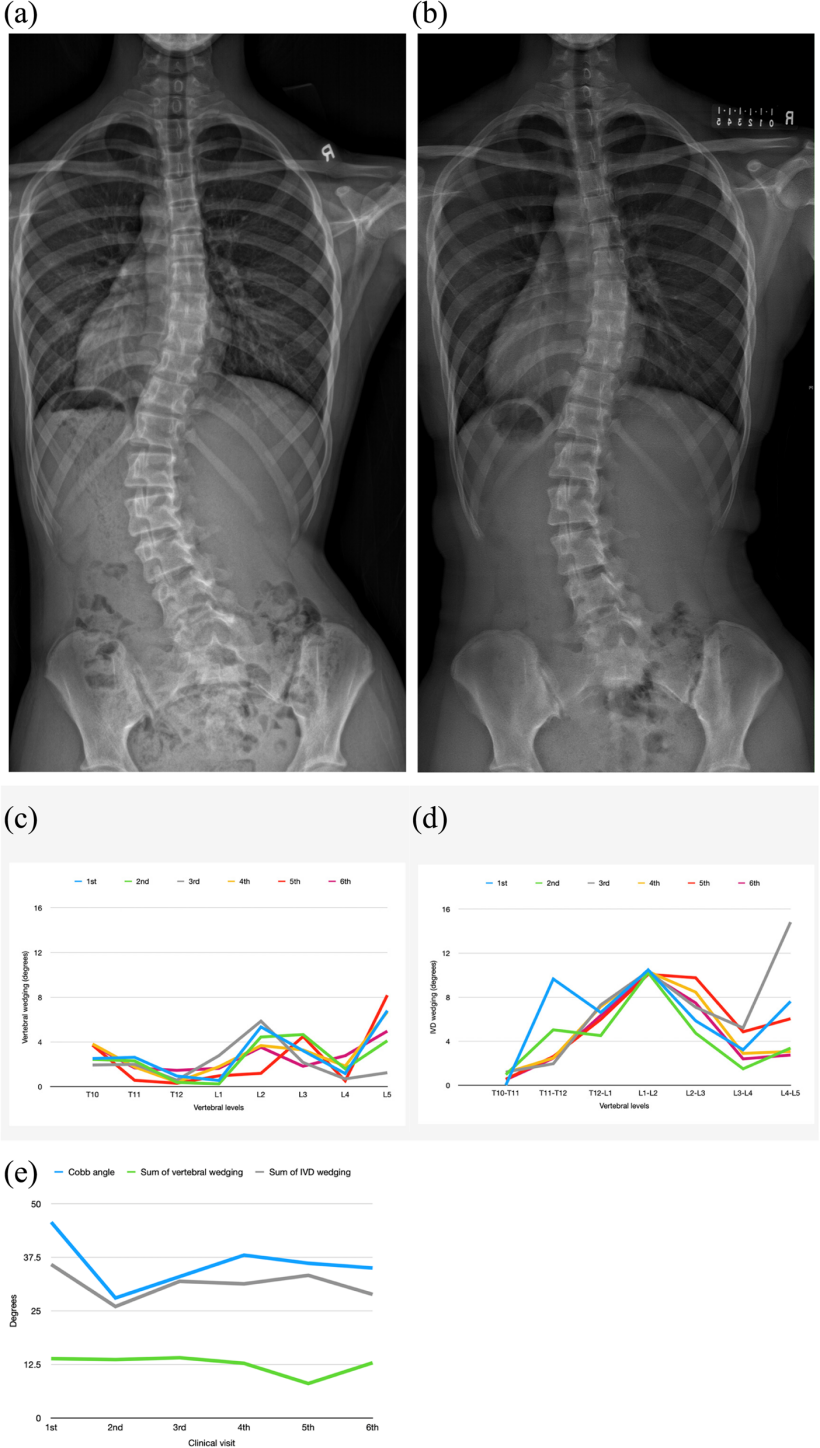
Non-progressed curves. **a** X-ray at the first clinical visit. T4/T11 = 25.2°. T11/L4 = 45.7°. **b** X-ray of the latest clinical visit. T4/T11 = 26°. T11/L4 = 35°. **c** Vertebral wedging of the curve T11/L4. The wedging at the apex (L2) increased by 0.5° at the 3rd clinical visit. **d** IVD wedging of the curve T11/L4. The wedging at the apex (L1/L2) was maintained at around 10°. **e** Summation of the wedging value of the lumbar curve. The sum of vertebral wedging decreased from 13.9° to 12.9° and that of IVD wedging decreased from 35.8° to 28.8°

20.8% and 16.7% of curves in the non-progression group and 11.4% and 8.6% of curves in the progression group had maximum sum of the entire curve’s vertebral and IVD wedging respectively at the first clinical visit. Thus, a larger percentage of non-progressed curves had a decreasing trend of the sum of wedging than progressed curves. The percentage decrease from the first visit to the latest visits was similar (< 1% difference) between vertebral wedging and IVD wedging in the progressed group. The percentage decrease of IVD wedging was 27% in the non-progressed curve which was larger than that of vertebral wedging (18.6%). Line graphs of curves (Fig. 10) having this condition in the non-progression group were assessed. There was no significant change (< 1°) in the wedging value at the apex or along the curve. This change of wedging was found in both vertebrae and IVD for a non-progressed curve.

Overall, 29% and 20% of curves in the progression group had a significant trend of vertebral and IVD wedging from the first to latest clinical visit. For non-progression group, only 4% of curves had a significant trend in IVD wedging.

### Intra- and inter-rater reliability

Fifteen radiographs were randomly selected from 5 subjects. Two observers independently measured the wedging and each dataset was measured twice with more than two weeks apart. ICC coefficients of vertebral wedging was from 0.77 to 0.92 and that of IVD wedging was from 0.72 to 0.90. The interclass correlation coefficient of vertebral wedging was from 0.78 to 0.90 and that of IVD wedging was from 0.84 to 0.94. Thus, both intraclass and interclass correlation achieved good reliability (Table 5).

**Table 5:** Intra- and inter-rater reliability

	Vertebral wedging	IVD wedging
Intra-rater reliability (intraclass correlation coefficient)	0.77 to 0.92	0.72 to 0.9
Inter-rater reliability (interclass correlation coefficient)	0.78 to 0.9	0.84 to 0.94

## Discussion

This study aimed to investigate the wedging pattern, sequence of wedging development, and the relationship between the change of wedging and curve progression in patients with AIS who received brace treatment with right-sided thoracic and/or left-sided lumbar curves. The results from this study indicated that the wedging pattern is specific to curve location. More vertebral wedging is identified in the thoracic curve and more IVD wedging is identified in the lumbar curve. IVD wedging can develop first followed by vertebral wedging or vice versa may occur and the sequence is linked to the spine location. A gradual increase in vertebral and IVD wedging of the curve was found in progressed curves and a significant difference in wedging between the first consultation and the latest was found in the progression group which had more significant trend in wedging during growth than in the non-progression group.

Previous studies found maximum wedging at the apex which decreased to the upper and lower vertebrae [6, 10, 16]. This pattern of wedging was found in most subjects of both groups for the major curve, thoracolumbar curve, and lumbar curve at the first and latest clinical visits. Vicious cycle theory proposes that minimal wedging in the vertebra may enhance wedging by producing an abnormal compressive force on the vertebral endplate based on the Hueter-Volkmann’s law [19]. Our results interestingly do not support this theory because wedging was found in the early stage of scoliosis of both progressed and non-progressed curves. Moreover, the value of wedging at the early stage of scoliosis could not differentiate between progression and non-progression groups. Thus, the current finding suggests that the initial presence of wedging cannot be used to classify a curve into progressive or non-progressive types.

The sum of vertebral wedging of the curve was larger than that of IVD wedging in the thoracic spine and the opposite was true for the lumbar curve. This suggests that the contribution of wedging is affected by the spinal location which corroborates with previous studies [6, 11, 13, 20]. However, the factors contributing to this segmental difference in wedging are still unknown. Possible factors include vertebrae size, tissues near the vertebrae, or spinal flexibility. The vertebral size is smaller in the thoracic region than in the lumbar region [21] and so the resultant action of the thoracic spine will be larger than that of the lumbar spine when the same compressive forces are applied. The back muscles are not uniform, and the rib cage is near the thoracic spine. Thus, the tissue composition near the vertebrae is not the same between the thoracic and lumbar curves which may lead to differences in wedging along the spine. Since an increase in Cobb angle was related to the reduced spinal range of motion [22] and the lumbar spine is more flexible than the thoracic spine, the flexibility level may affect the segmental development of wedging. It is important to understand the mechanism behind this as this may link to the pathogenesis of scoliosis.

After the pattern of wedging was identified, the sequence of wedging development was investigated. The results of this study suggest that vertebral wedging is developed first and followed by IVD wedging or vice versa may occur. Will et al. [23] reported that the increase in IVD wedging was largest in the early stage with increased vertebral wedging in the later stage. They concluded that curve progression started in the IVD followed by vertebral changes. However, this study used a different method to investigate the sequence of development via wedging pattern detection. As shown in figs. 6 and 7, wedging can be similar along the curve at the early stage of scoliosis. Afterwards, the wedging becomes maximum at the apex and decreases at the two ends. Thus, using wedging pattern detection to determine the development sequence is more accurate. The sequence of wedging development was found to be related to the spine location as vertebral wedging was developed first in the majority of thoracic curves but after IVD wedging in most of the lumbar curves. In relation to the previous point that vertebral wedging occurs more in the thoracic spine while IVD wedging occurs more in the lumbar spine, the mechanism of wedging development and the contribution of wedging to the curve magnitude are associated more with the location in the spine rather than an effect of curve progression.

When investigating the relationship between the change of wedging and curve progression, a larger percentage of curves in the progression group had an increasing trend in the sum of wedging of the entire curve from the first appointment to the latest follow-up than that of the non-progression group. On the other hand, there was a larger percentage of curves with a decreasing trend during growth in the non-progression group than in the progression group. This finding was consistent with previous studies whereby major curve Cobb angles were positively correlated with the increase in wedging [11] and increased ratio of the convex height divided by the concave height at the apex [24]. Both vertebral and IVD wedging had significant differences between the first consultation and the latest for the progression group. However, no significant change in wedging between two follow-ups was found in two previous studies [12, 13]. These studies did not consider the effect of curve type and maturity stages which may explain the differences with our study results. Thus, a clear difference in the change of wedging during growth between progressed and non-progressed curves is observed.

When comparing vertebral wedging to IVD wedging, the major curve of both groups had a larger sum of IVD wedging than vertebral wedging. For curves having an increasing trend in the sum of wedging during growth, the percentage increase in IVD wedging was more than vertebral wedging in the progressed group. The opposite however was observed in the non-progressed group. Fraser et al. found that the sum of vertebral wedging was larger than IVD wedging for the majority of curves [11]. Their study did not follow patients until brace weaning and this may explain the inconsistencies. The current study findings suggest that the sum of IVD wedging is more apparent in the major curve for both groups. The progressed curve has a higher possibility of increased overall IVD wedging as compared to the vertebrae. A small percentage of the non-progressed curves also has increasing trends of vertebral wedging compared to the IVD. Thus, the mechanism of curve development between the two groups may be different.

Spinal flexibility and in-brace correction have been used to predict curve progression [25, 26]. Our findings suggest that a gradual increase in wedging along the curve and a relatively larger change in IVD wedging than vertebral wedging represent a higher possibility of curve progression. Spinal flexibility, in-brace correction, and wedging should be measured to help clinicians better prognosticate outcomes. However, the relationship between the three parameters remains unclear. Future studies may focus on finding how one parameter affects the other to develop a more precise curve prediction model.

There are a few limitations of note. This study only included frontal plane radiographs and excluded the sagittal plane. Since the amount of sagittal wedging was smaller compared to the wedging in the coronal plane [13], this would be a small limitation. Manual measurement of vertebral endplates is a potential measurement error. The measurement may also be affected by vertebral rotation. Since the vertebral body is similar to a cylinder, the effect of vertebral rotation on wedging is limited. Furthermore, brace compliance was not measured in this study and its effect on wedging is unclear. Future study will require 3D imaging to study the effects of vertebral rotation. The sample size can also be increased to further validate our findings.

This was a comprehensive analysis of the entire curve pattern from every follow-up of patients with AIS. The major difference between the progressed and non-progressed curves was the change of wedging during growth. A significant change in wedging between the first consultation and the latest was found only in the progression group. Pattern and sequence of wedging were related to the location of the curve rather than the presence of curve progression. Future studies should focus on the reasons which cause segmental difference and factors affecting the gradual increase in wedging in the progressed curve by including patients in observation with different wedging in the early stage of scoliosis.

## Data Availability

Data can be shared upon contact with the correspondence author.
